# Evaluation of the effect of *Foeniculum vulgare* fruit and *Echium amoenum* flower combination on the severity of physical and psychological symptoms of premenstrual syndrome

**DOI:** 10.22038/AJP.2024.24208

**Published:** 2024

**Authors:** Simin Sadat Motevalli Haghi, Amirhossein Sahebkar, Jamshid Jamali, Roshanak Salari, Maliheh Motavasselian

**Affiliations:** 1 *Department of Persian Medicine, School of Persian and Complementary Medicine, Mashhad University of Medical Sciences, Mashhad, Iran*; 2 *Biotechnology Research Center, Pharmaceutical Technology Institute, Mashhad University of Medical Sciences, Mashhad, Iran*; 3 *Applied Biomedical Research Center, Mashhad University of Medical Sciences, Mashhad, Iran *; 4 *Department of Biotechnology, School of Pharmacy, Mashhad University of Medical Sciences, Mashhad, Iran*; 5 *Department of Biostatistics, School of Health, Social Determinants of Health Research Center, Mashhad University of Medical Sciences, Mashhad, Iran*; 6 *Department of Pharmaceutical Sciences in Persian Medicine, School of Persian and Complementary Medicine, Mashhad University of Medical Sciences, Mashhad, Iran*

**Keywords:** Premenstrual syndrome, Echium amoenum, Foeniculum vulgare, Herbal medicine, Phytoestrogens, Serotonin, Traditional Persian Medicine

## Abstract

**Objective:**

*Echium amoenum* (EA) and *Foeniculum vulgare* (FV) might be beneficial for the management of Premenstrual syndrome (PMS) due to their possible effects on sex hormones and neurotransmitters such as serotonin. This study aimed to investigate the effect of a combination of Echium and Fennel on the severity of physical and psychological symptoms of PMS.

**Materials and Methods:**

This triple-blinded, randomized, controlled trial was conducted on 80 women. The subjects were assigned to two groups of intervention (receiving EA 2 g/day and FV 1 g/day, in the second two weeks of the cycle, in two consecutive cycles) and placebo control. The data collection tools included the PSST, DRSP, and SF36 questionnaires.

**Results:**

Between-group differences in all subscales of DRSP were significant at one- and two-month time points (p<0.05). Bleeding volume was significantly increased in the intervention group, compared to the placebo group, two months after the intervention (p<0.05). Between-group comparison of the changes demonstrated significant differences in all subscales of SF36, except for limitations in usual role activities due to emotional problems (p=0.07).

**Conclusion:**

Consumption of EA and FV combination improved the quality of life in women with PMS and exerted favorable changes in PMS symptoms.

## Introduction

Premenstrual syndrome (PMS) is a frequently idiopathic condition characterized by a wide array of physical and psychological symptoms associated with reproductive age. These physical and behavioral symptoms, which appear in the luteal phase of the menstrual cycle (ovulation to the start of menstruation), negatively affect women's work and daily life (Bieber et al., 2015; Taylor, 2019; Dutta and Sharma, 2021). The criteria for premenstrual disorders have been mentioned in the American College of Obstetricians and Gynaecologists (ACOG), as well as the Diagnostic and Statistical Manual-fifth edition (DSM-5) (Association, 2013). The physical symptoms of PMS include swelling, headache, mastalgia, back pain, sleepiness or oversleeping, and its behavioral symptoms include fatigue, tension, anxiety, irritability and agitation, aggression, reduced short-term and long-term memory functions, and depression (Appleton, 2018; Gnanasambanthan and Datta, 2019).

Based on the World Health Organization reports, more than one hundred million women in Asia suffer from symptoms of menstruation. The prevalence of PMS symptoms varies in different cultures and geographical regions, being reported as 20-50% in most Western countries (Chiaramonte et al., 2017). Nonetheless, the estimated prevalence of PMS in Iran is 70.8% (Ranjbaran et al., 2017). The exact cause of PMS syndrome is not well known; however, investigations have referred to changes in the levels of sex hormones, changes of catecholamines, response to prostaglandins, and a decrease in the amount of central dopamine and serotonin (Zendehdel and Elyasi, 2018). Of these, serotonin has a critical importance owing to its major role in modulating mood, alertness, and circadian rhythms. Ample evidence suggests that serotonin decreases in PMS, leading to irritability, depression, mood changes, sleep disorders, aggression, and anxiety, as well as decreased pain tolerance threshold and concentration (Rapkin and Akopians, 2012).

The suggested treatments for PMS include selective serotonin reuptake inhibitors (SSRIs), alprazolam, Gonadotropin-releasing hormone (GnRH) agonists, oral contraceptives, exercise and relaxation techniques, diuretics, and surgery (Appleton, 2018; Ryu and Kim, 2015); nonetheless, none of these mentioned treatments is known as a standard treatment for PMS (Sadock, 2007). Furthermore, the severe side effects of some of these therapeutic approaches, as well as a failure to respond to treatment, and drug contraindications in some women, have highlighted the need to seek new treatment methods to control the symptoms of PMS. Phytotherapy studies have investigated the effect of various medicinal plants on the symptoms of PMS (Appleton, 2018; Maleki-Saghooni et al., 2018). Some evidence has demonstrated that 80% of Iranian women who suffer from PMS tend to use complementary and alternative medicine treatments (Babazadeh and Keramat, 2011). However, the efficacy of herbal treatments has not yet been recognized by authoritative texts in gynecology and psychiatry.

*Echium amoenum* (EA) and* Foeniculum vulgare *(FV) are two well-known native plants that have been widely used in Iran since ancient times. Some evidence illustrated that medicinal plants affect sex hormones and neurotransmitters, such as serotonin (Bawazir and Bokhary, 2016; Akbar, 2020; Karampoor et al., 2014). Fennel fruits contain alkaloids, carbohydrates, phytosterols, phenols, tannins, and flavonoids as non-volatile substances. The estrogenic, antispasmodic, analgesic, diuretic, anti-inflammatory, antimicrobial, antioxidant, and protective effects of FV have been confirmed in some studies (Al-Amoudi, 2017; Gori et al., 2012). Unlike the western samples of *Borago officinalis*, some evidence suggested that the flowers of Persian *Echium amoenum* lack alkaloids, tannins, and cyanogenic glycosides; moreover, they contain different amounts of flavonoids, saponins, and unsaturated sterols (Amirghofran et al., 2000). 

In general, the total level of serotonin and the response of serotonin to estrogen and progesterone hormones are lower in people with PMS compared with normal people. Fennel has a phytoestrogen component, and *Echium* has serotonergic effects; therefore, it seems that the combination of the aforementioned plants has positive effects on the PMS. Nonetheless, clinical studies conducted on these plants are not enough and sometimes contradictory results have been observed in this regard. 

Due to the absence of serious side effects for Fennel and Echium in usual doses, the high prevalence of PMS among Iranian women, considering that the changes in sex hormones and neurotransmitters such as serotonin are the possible cause of PMS, as well as the interaction of serotonin and sex hormones, this idea was formed that a combination of phytoestrogen and serotonergic plants could be used to reduce the symptoms of PMS. The present study aimed to assess the effect of the combination of FV and EA on the severity of physical and psychological symptoms of PMS. 

## Materials and Methods

This triple-blinded, randomized, placebo-controlled trial was conducted on women referring to traditional medicine specialists in Mashhad, Iran, in 2021.

**Inclusion and exclusion criteria**

The inclusion criteria entailed an age range of 18-35 years, regular menstrual cycle in the last six months, 3-9 days of menstrual bleeding, 24-35-day menstrual cycle, and receiving a minimum score of 19 in Premenstrual Symptoms Screening Tool (PSST) questionnaire. On the other hand, the non-inclusion criteria were as follows: pregnancy, lactation, a history of mental illnesses, surgery, estrogen-dependent cancers, diabetes, thyroid, hypertension, liver or kidney diseases, an experience of grief in the last six months, use of anti-anxiety or anti-depression drugs in the last three months, use of drugs containing estrogen or progesterone during the previous three months, use of herbal drugs, vitamins or mineral supplements in the last month, or a history of allergy to FV or EA. 

**Research tools**

**Premenstrual symptoms screening tool (PSST)**

This scale includes 19 items in two parts. The first part contains 14 emotional, physical, and behavioral symptoms, while the second part encompasses 5 questions that measure the effect of these symptoms on people's lives (mild: scores between 0-19, moderate: scores between 19 and 28, and severe: scores above 28). The reliability of the Persian version of this tool was confirmed with Cronbach's alpha values of 0.9. The values of the content validity ratio and content validity index were obtained at 0.7 and 0.8, respectively, indicating the appropriate content validity of the questionnaire (Mirghafourvand et al., 2015). 

**Daily record of severity of problems (DRSP)**

This 21-item form, which contains 11 domains (depression, anxiety, lability, anger, interest in activities, concentration, lethargy, appetite, sleep, control, and physical symptom), is used to diagnose PMS and premenstrual disorder. Each item is rated from 1 (not at all) to 6 (extreme) (Endicott et al., 2006). The validity and reliability of the Persian version of this form have been investigated in the study by Izadi (Izadi, 2010).

**Short-Form health survey questionnaire (SF36)**

This 36-item scale is a standard questionnaire used to assess the quality of life in eight main domains of physical functioning, social functioning, physical role-playing, emotional role-playing, mental health, vitality, pain, and general health. The subject's score in each domain varies from 0 to 100, with a higher score signifying a better quality of life. The validity and reliability of this questionnaire have been confirmed in the Iranian population (Montazeri et al., 2005). 

**Preparation and standardization of a combination of** ***Foeniculum vulgare*** **and** ***Echium amoenum***

Echium flowers used in this study were from Mazandaran, north of Iran, and fennel fruits were obtained from Razavi Khorasan province, Iran. The plants were registered with herbarium codes (E-1246 FUMH for EA and E-1247 FUMH for FV) in the herbarium center of the Ferdowsi University of Mashhad. After purchase, the plants were separately washed, dried, and then ground. Finally, the required dose for each person in one month was measured and after mixing, the capsules were filled and placed in the container.

 FV and EA hydroalcoholic extract was standardized considering the content of phenolic compounds. In the first step, Folin-Ciocalteu reagent (100 µl) and 1 mol/L sodium carbonate solution (300 µl) were added to a sample of 20 µl of the plant (10 mg/ml) or gallic acid as standard (50-500 mg/L) and mixed thoroughly. Approximately 2 ml of solvent (deionized water) was added and the contents were mixed using a vortex. The absorbance was measured at 765 nm by spectrometer two hours later. Finally, a standard curve was drawn to explain the gallic acid and the content of the phenolic compound of the FV and EA extract, as milligrams of gallic acid equivalents (Hooshmand et al., 2021). The content of total phenols in the extract of the combination of *F. vulgare* and *E. amoenum* was 42 mg gallic acid equivalent per gram of the crude extract. 

**Study design**

The sample size was calculated at 36 cases in each group considering α=0.05 and β=0.2 using the Power Analysis & Sample Size (PASS) Software (Delaram and Heydarnejad, 2011) and the following formula: 

$n=\frac{{\left(Z_{1-\frac{a}{2}}+Z_{1-\beta }\right)}^{2}(S_{1}^{2}+S_{2}^{2})}{{\left({\bar{x}}_{2}-{\bar{x}}_{1}\right)}^{2}}=\frac{{\left(1.96+0.84\right)}^{2}(22.4^{2}+16.1^{2})}{{\left(25.5-38.7\right)}^{2}}$

 A total of 40 cases were included in each group to consider possible sample attrition and increase the power of the test. Sampling was done from 4 traditional medicine outpatient treatment centers in Mashhad: Traditional Medicine Clinic of Imam Reza Hospital, Iranian Center, Tous Heart Center, Hikmat Center. The women who complained of PMS symptoms filled out the personal profile form and the premenstrual syndrome screening tool for initial diagnosis. Eligible participants completed the Daily Record of Severity of Problems (DRSP) for two consecutive months to diagnose PMS. After a diagnosis, 80 subjects were randomly selected and completed the Quality of Life questionnaire.

Permuted block technique was used to assign participants to study groups by random allocation software. The subjects were divided into two groups: intervention and control. The patients in the intervention group received capsules with a dose of EA 2 g and FV 1 g per day three times per day (morning, noon, and night) with a glass of lukewarm water in the second two weeks of the cycle, during two consecutive cycles. The patients in the control group received capsules containing starch with the same conditions. Placebo and herbal capsules were distributed in the same shape, packaging, and number. In this study, based on previous research and sources of Traditional Persian medicine, a daily dose of EA 2 g and FV 1 g was selected (Aghili Alavi Shirazi, 1881; Delaram and Heydarnejad, 2011; Azam Khan, 2004; Pazoki et al., 2016; Akbar, 2018). In traditional medical and pharmaceutical books, the combination of these two herbs is has been recommended (Aghili Alavi Shirazi, 1881; Avicenna et al., 1999; Azam Khan, 2004). The intervention period was two menstrual cycles. During these two cycles, the DRSP questionnaire was completed to compare changes in patients' symptoms. A channel was formed on WhatsApp, and participants joined it. The completion of forms was explained through an educational video and audio file. Patients could send their questions to the admin in private messages. To ensure that the capsules were consumed and the form was completed, they were reminded on the channel every morning, noon, and evening, and the patients were contacted every week. Finally, changes in the severity of symptoms and quality of life were compared between the two groups. 

**Figure 1:**
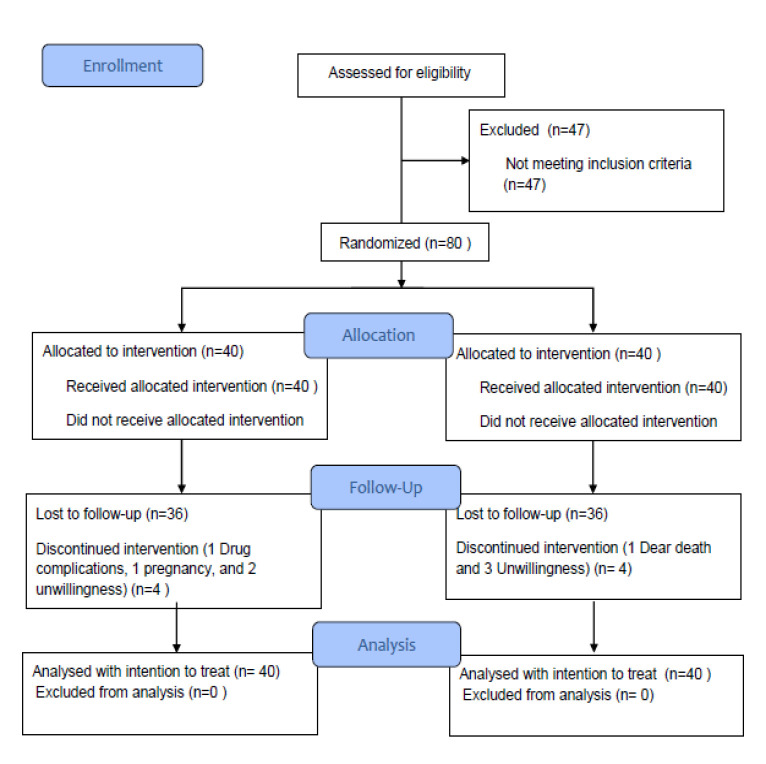
CONSORT flow diagram of study

The patients who received psychiatric and hormonal treatments, herbal medicines, vitamins, and mineral supplements, or those who experienced a stressful event such as death of a loved one, marriage, or pregnancy during the study, were removed. Moreover, the patients who were not willing to continue treatment or exhibited adverse side effects as a result of consumption of FV and EA were excluded from the study. CONSORT 2010 flow diagram presents the sample selection process in the study (Figure 1). Finally, two groups were compared in terms of SF36 and DRSP scores. 

**Ethical considerations**

The present study was extracted from a Ph.D dissertation in traditional Persian medicine (Code number: 981613). The research proposal has been approved by the Ethics Committee of Mashhad University of Medical Sciences (Ethics approval No. IR.MUMS.REC.1399278) and completed in accordance with Helsinki Declaration guidelines. Consent forms were obtain from all patients. To ensure the confidentiality of the data, it was coded and included in the checklist. The objectives of the study, as well as the method and steps of the study, were explained to all participants. All patients were assured that they could withdraw from the study at any time. The trial protocol was registered with the ID: IRCT20200530047600N1.

**Statistical analysis**

The data were analyzed in SPSS software (version 23). A total of 80 participants were included in the intention-to-treat analyses. In this study, the Intention-to-Treat (ITT) method was used to analyze the data. Therefore, all individuals who had received the intervention, At least once,were included in the final analysis. The normality condition of the quantitative variables was investigated by using the Kolmogorov–Smirnov test. The Chi-square test was used to compare qualitative variables between the two groups. An Independent t-test or Mann-Whitney U test was used to compare differences in quantitative variables between intervention and placebo groups, as appropriate. Moreover, paired T-test or its nonparametric equivalent, the Wilcoxon test, was used to compare the quantitative variables before and after the intervention. A p value less than 0.05 was considered statistically significant.

## Results

In general, 80 PMS women with a mean age of 28.59±4.76 years entered in this study. The mean age of participants in the intervention and control groups were 30.13±4.14 and 27.05±4.9 years, respectively. The body mass index (BMI) scores were 22.86±3.71 and 24.79±4.79 in the intervention and control groups, respectively. Moreover, the mean dysmenorrhea scores were 2.80±2.18 and 5.5±1.72 in the intervention and control groups, respectively. The two groups were not matched in terms of age (Z=-2.77; p=0.005), BMI (Z=-1.97; p=0.04), or dysmenorrhea (Z=-5.28; p<0.005). Table 1 presents the comparison of other demographic information in the two groups. Based on the obtained result, the two groups were matched in terms of education level and marriage status (p>0.05); however, they were not matched in terms of occupational status or having children (p<0.005).

**Table 1:** Comparison of the demographic information in the two groups

Categorical variables		Intervention		Control				χ^2^	p-value
		N	%	N	%	N	%		
Education level	Diploma and below	22	55	19	47.5	41	51.2	0.58**	0.890
	Bachelor's degree	14	35	17	42.5	31	38.8		
	Master's degree	4	10	4	10	8	10		
Occupational status	Employed	20	50	11	27.5	31	38.8	4.26*	0.030
	Housewife	20	50	29	72.5	49	61.3		
Marriage status	Married	27	67.5	19	47.5	46	57.5	3.27*	0.070
	Single	13	32.5	21	52.5	34	42.5		
Having child	Yes	25	62.5	9	22.5	34	42.5	13.09*	<0.005
	No	15	37.5	31	77.5	46	57.5		

* Chi-square test. ** Fisher's exact test

Table 2 illustrates the SF36 scores in women with PMS before and after the intervention in the two groups. The obtained results pointed out that intergroup changes between the two groups were significantly different in all subscales of SF36, except for limitations in usual role activities due to emotional problems (p=0.07). The Wilcoxon test showed that all subscales of SF36 were significantly improved in the intervention group (p<0.05), whereas no improvement was observed in the control group.

 (p>0.05). The total score of SF36 was significantly improved after the intervention in intervention group (Z=-5.304; p<0.005), while it was not changed in the control group (Z=-0.43; p=0.66), signifying an improvement in the quality of life after the consumption of FE and EA combination. 

The comparison of the two groups before and one month after the intervention in terms of DRSP subscales is displayed in Table 3. Based on the obtained results, intergroup changes in all subscales of DRSP were significantly different between the two groups (p<0.05). The Wilcoxon test demonstrated that the DRSP score was significantly improved one month after the intervention in both groups in terms of some subscales, including depression, anxiety, lability, anger, lethargy, appetite, control, and physical symptom (p<0.05). Nevertheless, the subscales of interest in activities, concentration, sleep were improved one month after the intervention only in intervention group (p<0.05). The total score of DRSP was significantly improved in both groups (p<0.05). Moreover, the bleeding volume was increased one month after the intervention in intervention group compared to the placebo group (p<0.05).

**Table 2:** SF36 score in women with PMS before and after intervention in the two groups

Variables		Intervention group	Control group	Test statistics	Intergroups p-value
		Mean± SD	Mean± SD		
Physical function	Before intervention	76.71±14.11	78±21.6	1.02*	0.305
	After intervention	88.65±10.58	78.03±22.68	1.33*	0.180
	Intergroup changes	12.43±8.63	0.66±3.89	-6.52*	<0.005
	Z	-4.89^c^	1.040^c^		
	P-value	<0.005	0.29		
Role-physical	Before intervention	51.97±31.51	55.63±28.02	-.406*	0.680
	After intervention	83.78±23.73	55.28±27.38	-4.25*	<0.005
	Intergroup changes	32.43±21.14	-2.61±7.72	-7.14*	<0.005
	Z	-5.09^c^	-1.89^c^		
	P-value	<0.005	0.059		
Role-emotional	Before intervention	79.82±28.52	41.67±39.76	-4.26*	<0.005
	After intervention	92.79±15.98	42.18±39.95	-5.48*	<0.005
	Intergroup changes	13.51±31.88	0.96±5.42	-1.77*	0.070
	Z	-2.5^c^	-1.342^d^		
	P-value	0.01	0.18		
Vitality	Before intervention	55.39±11.41	49±16.61	-1.71*	0.080
	After intervention	70.95±10.2	50.26±17.44	-5.17*	<0.005
	Intergroup changes	16.08±11.06	1.71±6.90	-5.37*	<0.005
	Z	-5.068^c^	-1.658^d^		
	P-value	<0.005	0.09		
Mental health	Before intervention	59.79±10.76	57.10±8.69	-1.96*	0.050
	After intervention	71.78±8.48	54.42±12.90	-5.43*	<0.005
	Intergroup changes	12.32±10.64	-2.21±8.14	-5.704*	<0.005
	Z	-5.042^c^	-1.696^c^		
	P-value	<0.005	0.09		
Social functioning	Before intervention	69.74±12.88	57.81±9.25	-4.37*	<0.005
	After intervention	85.14±9.70	56.91±12.23	-7.01*	<0.005
	Intergroup changes	16.22±13.46	-0.33±6.81	-5.607*	<0.005
	Z	-4.624^c^	-0.302^c^		
	P-value	<0.005	0.76		
Bodily pain	Before intervention	63.95±14.05	54.63±13.92	-2.77*	0.006
	After intervention	77.43±17.63	54.21±14.94	-5.34*	<0.005
	Intergroup changes	14.19±16.17	0.59±10.42	-4.36*	<0.005
	Z	-4.153^c^	-0.095^d^		
	P-value	<0.005	0.92		
General health	Before intervention	54.47±11.90	56.38±12.35	-0.36*	0.71
	After intervention	66.08±10.28	55.26±11.68	-3.8*	<0.005
	Intergroup changes	12.30±8.71	-0.53±5.79	-6.56*	<0.005
	Z	-5.197^c^	-0.275^c^		
	P-value	<0.005	0.78		
Total score	Before intervention	64.21±11.30	60.07±14.05	1.42**	0.15
	After intervention	79.96±10.73	59.57±15.58	-5.56*	<0.005
	Intergroup changes	16.32±9.16	-0.64±3.54	-7.31*	<0.005
	Z	-5.304^c^	-0.433^c^		
	P-value	<0.005	0.66		

**t-test. *Mann–Whitney U test. b. Wilcoxon Signed Ranks Test. c. Based on negative ranks. d. Based on positive ranks

**Table 3:** The comparison of two groups before and after one month of intervention in terms of daily record of severity of problems subscales.

Variables		Intervention group	Control group	Test statistics	Intergroups p-value
		Mean± SD	Mean± SD		
Depression	Before intervention	11.75±1.79	10.40±1.45	-3.63*	<0.005
	After intervention	5.92±1.08	9.96±1.74	-7.48*	<0.005
	Intergroup changes	-5.82±2.08	-0.44±0.98	-7.53*	<0.005
	Z	-5.51^d^	-2.693^c^		
	P-value	<0.005	0.007		
Anxiety	Before intervention	4.11±0.66	3.55±0.57	4.12**	<0.005
	After intervention	2.05±0.59	3.40±0.63	-9.97**	<0.005
	Intergroup changes	-2.07±0.85	-0.15±0.29	-7.59*	<0.005
	Z	-5.512^d^	-3.183^c^		
	P-value	<0.005	0.001		
Lability	Before intervention	7.62±1.11	7.05±1.53	1.909**	0.06
	After intervention	4.10±1.00	6.75±1.60	-6.603*	<0.005
	Intergroup changes	-3.52±1.41	-0.30±0.57	-7.42*	<0.005
	Z	-5.512^d^	-3.329^c^		
	P-value	<0.005	0.001		
Anger	Before intervention	7.79±1.12	7.24±0.79	2.52**	0.01
	After intervention	4.14±1.08	6.87±0.94	-7.06*	<0.005
	Intergroup changes	-3.64±1.52	-0.37±0.54	-7.38*	<0.005
	Z	-5.511^d^	-4.417^c^		
	P-value	<0.005	<0.005		
Interest in activities	Before intervention	4.07±0.65	3.52±0.96	-2.86*	0.004
	After intervention	1.98±0.65	4.14±4.89	-5.88*	<0.005
	Intergroup changes	-2.09±0.85	0.61±4.78	-7.31*	<0.005
	Z	-5.444^d^	-1.812^c^		
	P-value	<0.005	0.07		
Concentration	Before intervention	3.53±0.68	3.19±0.36	-2.76*	0.006
	After intervention	1.73±0.50	3.12±0.40	-7.26*	<0.005
	Intergroup changes	-1.81±0.83	-0.07±0.34	-7.38*	<0.005
	Z	-5.444^d^	-1.048^c^		
	P-value	<0.005	0.29		
Lethargy	Before intervention	4.06±0.61	3.66±0.57	-2.82*	0.005
	After intervention	1.97±0.55	3.41±0.75	-6.48*	<0.005
	Intergroup changes	-2.09±0.77	-0.25±0.51	-7.29*	<0.005
	Z	-5.512^d^	-4.110^c^		
	P-value	<0.005	<0.005		
Appetite	Before intervention	7.29±1.51	6.44±2.44	1.88**	0.06
	After intervention	3.95±1.81	6.33±2.36	-4.46*	<0.005
	Intergroup changes	-3.34±2.42	-0.11±0.41	-6.29*	<0.005
	Z	-5.236^d^	-2.582^c^		
	P-value	<0.005	0.01		
Sleep	Before intervention	7.33±1.30	6.24±1.23	-3.807*	<0.005
	After intervention	4.18±1.04	6.15±1.34	-5.84*	<0.005
	Intergroup changes	-3.16±1.43	-0.09±0.45	-7.42*	<0.005
	Z	-5.511^d^	-1.333^c^		
	P-value	<0.005	0.18		
Control	Before intervention	7.93±1.30	6.28±1.24	5.805**	<0.005
	After intervention	4.03±0.72	6.11±1.21	-6.709*	<0.005
	Intergroup changes	-3.89±1.22	-0.17±0.47	-7.62*	<0.005
	Z	-5.511^d^	-2.662^c^		
	P-value	<0.005	0.008		
Physical Symptom	Before intervention	13.65±3.03	12.84±1.92	1.43**	0.15
	After intervention	7.62±1.96	12.46±1.84	-7.01*	<0.005
	Intergroup changes	-6.04±2.70	-0.37±0.81	-7.24*	<0.005
	Z	-5.484^d^	-2.714^c^		
	P-value	<0.005	0.007		
Bleeding volume	Before intervention	1.74±0.31	1.99±0.46	-2.11*	0.03
	After intervention	1.95±0.44	1.99±0.46	-0.15*	0.87
	Intergroup changes	0.21±0.39	0	-3.13*	0.002
	Z	-3.138^c^	0		
	P-value	0.002	>0.99		
Total score	Before intervention	79.13±10.16	70.40±10.04	3.86**	<0.005
	After intervention	41.67±7.60	68.68±12.59	-7.26*	<0.005
	Intergroup changes	-37.46±11.48	-1.71±6.97	-7.66*	<0.005
	Z	-5.511^d^	-3.347^c^		
	P-value	<0.005	0.001		

**t-test. *Mann–Whitney U test. b. Wilcoxon Signed Ranks Test. c. Based on negative ranks. d. Based on positive ranks

Table 4 compares the two groups before and two month after initiation of the treatment in terms of DRSP subscales. Based on this table, intergroup changes were significantly different in all subscales of DRSP between the two groups (p<0.05). According to the Wilcoxon test, DRSP subscales, including anxiety, lability, anger, interest in activities, lethargy, and physical symptom, were significantly improved one month after intervention in both groups (p<0.05), while the subscales of depression, concentration, appetite, sleep, and control were improved only in intervention group (p<0.05). The total score of DRSP was significantly improved in both groups two months after the intervention (p<0.05). Finally, bleeding volume was significantly increased in the intervention group two months after the intervention (p<0.05), which could be considered one of the side effects of the herbal preparation. 

**Table 4:** The comparison of the two groups before and after two months of intervention in terms of Daily Record of Severity of Problems subscales

Variables		Intervention group	Control group	Test statistics	Intergroups p-value
		Mean±SD	Mean±SD		
Depression	Before intervention	11.75±1.79	10.4±1.45	-3.63*	<0.005
	Two months after intervention	4.87±1.02	9.95±1.85	-7.21*	<0.005
	Intergroup changes	-7.01±1.81	-0.30±0.94	-7.301*	<0.005
	Z	-5.232^d^	-1.914^c^		
	p-value	<0.005	0.056		
Anxiety	Before intervention	4.11±0.66	3.55±0.57	4.12**	<0.005
	Two months after intervention	1.62±0.48	3.4±0.63	-6.98*	<0.005
	Intergroup changes	-2.52±0.78	-0.14±0.38	-7.202*	<0.005
	Z	-5.233^d^	-2.096^c^		
	p-value	<0.005	0.036		
Lability	Before intervention	7.62±1.11	7.05±1.53	1.909**	0.06
	Two months after intervention	3.40±0.83	6.70±1.58	-6.91*	<0.005
	Intergroup changes	-4.23±1.12	-0.23±0.55	-7.31*	<0.005
	Z	-5.233^d^	-2.708^c^		
	p-value	<0.005	0.007		
Anger	Before intervention	7.79±1.12	7.24±0.79	2.52**	0.01
	Two months after intervention	3.34±0.88	6.88±0.92	-7.09*	<0.005
	Intergroup changes	-4.48±1.44	-0.31±0.5	-7.005*	<0.005
	Z	-5.16^d^	-3.245^c^		
	p-value	<0.005	0.001		
Interest in activities	Before intervention	4.07±0.65	3.52±0.96	-2.86*	0.004
	Two months after intervention	1.64±0.55	3.33±0.87	-6.605*	<0.005
	Intergroup changes	-2.48±0.64	-0.14±0.35	-7.33*	<0.005
	Z	-5.233^d^	-2.750^c^		
	p-value	<0.005	0.006		
Concentration	Before intervention	3.53±0.68	3.19±0.36	-2.76*	0.006
	Two months after intervention	1.43±0.42	3.12±0.37	-6.96*	<0.005
	Intergroup changes	-2.13±0.75	-0.06±0.27	-7.07*	<0.005
	Z	-5.162^d^	-1.045^c^		
	p-value	<0.005	0.29		
Lethargy	Before intervention	4.06±0.61	3.66±0.57	-2.82*	0.005
	Two months after intervention	1.67±0.55	3.44±0.68	-6.86*	<0.005
	Intergroup changes	-2.36±0.8	-0.19±0.28	-7.04*	<0.005
	Z	-5.162^d^	-3.605^c^		
	p-value	<0.005	<0.005		
Appetite	Before intervention	7.29±1.51	6.44±2.44	1.88**	0.06
	Two months after intervention	3.00±0.99	6.27±2.29	-5.97*	<0.005
	Intergroup changes	-4.49±1.86	-0.05±0.49	-7.35*	<0.005
	Z	-5.232^d^	-1.569^c^		
	p-value	<0.005	0.11		
Sleep	Before intervention	7.33±1.30	6.24±1.23	-3.807*	<0.005
	Two months after intervention	3.53±0.82	6.23±1.20	-6.56*	<0.005
	Intergroup changes	-3.94±1.37	<0.01±0.36	-7.17*	<0.005
	Z	-5.232^d^	-0.278^c^		
	p-value	<0.005	0.78		
Control	Before intervention	7.93±1.30	6.28±1.24	5.805**	<0.005
	Two months after intervention	3.30±0.85	6.16±1.21	-6.89*	<0.005
	Intergroup changes	-4.63±1.33	-0.05±0.34	-7.31*	<0.005
	Z	-5.232^d^	-1.202^c^		
	p-value	<0.005	0.22		
Physical Symptom	Before intervention	13.65±3.03	12.84±1.92	1.43**	0.15
	Two months after intervention	7.60±2.21	12.53±1.71	-10.59**	<0.005
	Intergroup changes	-6.45±2.63	-0.19±0.73	-6.89*	<0.005
	Z	-5.216^d^	-1.970^c^		
	p-value	<0.005	0.049		
Bleeding volume	Before intervention	1.74±0.31	1.99±0.46	-2.11*	0.03
	Two months after intervention	1.97±0.41	2.00±0.48	-0.59*	0.55
	Intergroup changes	0.2±0.4	0±0	-2.78*	0.005
	Z	-2.850^c^	0		
	p-value	0.004	>0.99		
Total score	Before intervention	79.13±10.16	70.40±10.04	3.86**	<0.005
	Two months after intervention	35.39±6.75	68.00±10.05	-7.15*	<0.005
	Intergroup changes	-44.7±9.07	-1.67±3.65	-7.29*	<0.005
	Z	-5.232^d^	-2.781^c^		
	p-value	<0.005	0.005		

**t-test. *Mann–Whitney U test. b. Wilcoxon Signed Ranks Test. c. Based on negative ranks. d. Based on positive ranks. e. The sum of negative ranks equals the sum of positive ranks

## Discussion

As evidenced by the results of the present study, the consumption of FV and EA combination improved the quality of life in women with PMS. The use of these herbal products led to stable changes in depression, concentration, appetite, sleep, and control in PMS patients, while anxiety, lability, anger, lethargy, physical symptoms, and interest in activities were improved as the placebo effects. Moreover, the total score of DRSP was improved in both groups one and two months after the intervention. Nonetheless, the difference between the groups in all DRSP subscales was also significant at one and two months. We found no similar studies assessing the positive effects of FV and EA combination on the quality of life and management of symptoms of PMS. Therefore, we did not have access to sufficient information to compare with the present findings. However, some studies assessed the effect of these herbal medicines alone or in combination with different types of exercise in women suffering from PMS. 

Some evidence pointed out that Fennel extract could relieve dysmenorrhea (Omidali, 2015; Seidlova-Wuttke and Wuttke, 2017; Torkzahrani et al., 2007). However, the effect of Fennel on the psychological symptoms of the menstrual condition has been investigated in a few studies (Salehi et al., 2019). The impact of Fennel on dysmenorrhea is probably due to its inhibitory effects on prostaglandin E2 and oxytocin production (Ostad et al., 2001). It appears that Fennel may exert its effects through hormonal changes in women experiencing PMS symptoms (Delaram and Heydarnejad, 2011). 

The positive effects of Fennel on decreasing the severity of PMS symptoms have been confirmed in previous studies (Delaram et al., 2011; Pazoki et al., 2016). In this regard, Pazoki et al. compared the effect of aerobic exercises in combination with Fennel on PMS and confirmed the effects of Fennel on reducing the symptoms of PMS (Pazoki et al., 2016). In a similar vein, Kazemi et al. reported that resistance training along with Fennel reduced the severity of emotional-behavioral and physical symptoms of PMS in non-athletic adolescent female students. Moreover, more improvement was reported in physical and psychological symptoms than in emotional and behavioral symptoms (Kazemi et al., 2022). These findings have been confirmed in other similar studies (Delaram and Heydarnejad, 2011; Omidali, 2015; Ostad et al., 2001). 

Another study by Delaram et al. pointed out that stress, depression, and somatic symptoms improved after the consumption of Fennel extract every eight hours for three days, while it had no effect on excitement and bloating. Authors confirmed the stable effects of Fennel extract on the PMS for at least three cycles after treatment (Delaram et al., 2001). Nevertheless, the results of our study confirmed the effects of the FV and EA combination on both physical and psychological symptoms of PMS. Moreover, a stable change has been reported in mood symptoms, including depression, concentration, appetite, sleep, and control. Since no information is available to verify the findings of the present study, it is recommended to conduct more studies in this regard.

The effect of Fennel may be secondarily to its spasmolytic effect exerted due to the structural similarity of anethole present in Fennel to dopamine, binding to dopamine receptors and reducing pain (Bekhradi and Zafari, 2012). Moreover, studies have demonstrated that effective substance of Fennel extract is capable of inhibiting the contractions of uterine smooth muscle caused by oxytocin and prostaglandin, and in this way, it can reduce pain. In addition, some findings have suggested that the antispasmodic effect of Fennel are exerted by inhibiting contractions caused by acetylcholine and histamine (Bekhradi and Zafari, 2012). 

Clinical studies on *Echium amoenum* confirmed the effectiveness of this plant in depression and anxiety disorders (Azizi et al., 2018). Furthermore, EA has been used by naturopathic practitioners to regulate the metabolism and the hormonal system (Amirghofran, 2010) and manage anxiety and menopausal symptoms, such as hot flashes (Miraj and Kiani, 2016). The functional mechanism of EA seemingly depends on a fatty acid of gamma-linolenic acid (GLA), which has some anti-inflammatory and antioxidant effects (Miraj and Kiani, 2016). The role of GLA in the decrease of the severity and duration of PMS symptoms has been confirmed (Watanabe et al., 2005). Moreover, there is evidence concerning the efficacy of herbal medicine rich in GLA in reducing the severity of PMS symptoms (Mahboubi, 2019). Only one study has evaluated the therapeutic effect of EA on the management of PMS symptoms (both emotional symptoms and physical symptoms), and it pointed to a reduction in the symptoms of PMS after receiving 450 mg capsules of EA per day for two consecutive cycles (Farahmand et al., 2020). Gama et al. indicated a reduction in PMS symptoms after treatment with *Borago officinalis* in 95.4% of patients with no serious adverse events, the most common of which were related to the digestive/gastrointestinal tract (Gama et al., 2014). 

High-sensitivity C-reactive protein levels, altering inflammation from a physiological to a pathological state, increased oxidative stress, and decreased antioxidant capacity were observed during PMS, exacerbating its symptoms.(Duvan et al., 2011; Gold et al., 2016). The anti-inflammatory, analgesic, antioxidant, anti-anxiety, and psychiatric effects of EA support its beneficial effects on decreasing the severity of PMS symptoms (Sayyah et al., 2009; Sayyah et al., 2012) 

The effects of herbal medicine on the reduction of PMS symptoms could be explained by improving patients' self-control over their lives (Bryant et al., 2005; Khayat et al., 2015). This was corroborated by the placebo’s psychological effects, which contributed to a decrease in the severity of PMS symptoms (Akbarzadeh et al., 2015). In this regard, patient acceptance and tolerability aspects are crucial in the treatment of women who experience PMS symptoms (Ostad et al., 2001). The present study points to the effectiveness of using a placebo for psychological symptoms of PMS. The total score of DRSP was improved in both intervention and control groups after the intervention.

While it appears that a combination of Fennel and Echium could potentially serve as an effective treatment for PMS symptoms, the standard dosage, concentration, and treatment duration remain unclear due to the heterogeneity of findings. This makes it challenging to determine the optimal dosage for this product’s effect on PMS symptoms (Salehi et al., 2019). Furthermore, we lack sufficient data regarding the optimal usage of these herbal medicines. Some findings demonstrated that Fennel extract is more effective than Fennel essence since it is derived from the seed (Torkzahrani et al., 2007). 

The current study may introduce a novel treatment for managing menstruation symptoms, underscoring the importance of integrating traditional medicine with neuroscience. 

Among the notable limitations of this study, we can refer to the COVID-19 pandemic, which to some extent, limited the availability of a homogeneous sample. The unmatched groups in terms of age, BMI, dysmenorrhea, occupational status, and having children may have affected the accuracy of our data. Moreover, due to long time of the study and the possibility of the effect of the season on mood symptoms, the accuracy of our data may be affected. Furthermore, due to the possibility of increased bleeding following the use of this combination, it should be administered considering the patient's condition. We suggest to conduct multicenter clinical trials to determine the effectiveness of Fennel against symptoms of PMS, as well as the appropriate dose and duration of treatment, with adjusting confounding factors.

It seems that *Echium amoenum* in combination with Fennel could be used as a good treatment for women with PMS. The present study revealed that EA and FV combination reduced the severity of premenstrual syndrome and increased the quality of life in patients with PMS. Moreover, the use of herbal medicines could lead to stable changes in depression, concentration, appetite, sleep, and control in patients with PMS. However, it should be considered that bleeding volume increases following the use of EA and FV combination. 

## References

[1] Aghili Alavi Shirazi MHIMH (1881). Al-makhzan al-adavieh: dictionary of traditional medicines.

[2] Akbar S (2018). Fennel (Foeniculum vulgare Mill ): A common spice with unique medicinal properties. Ann Complement Altern Med.

[3] Akbar S (2020). Borago officinalis l.(boraginaceae). handbook of 200 medicinal plants.

[4] Akbarzadeh M, Dehghani M, Moshfeghy Z, Emamghoreishi M, Tavakoli P, Zare N (2015). Effect of melissa officinalis capsule on the intensity of premenstrual syndrome symptoms in high school girl students. Nurs Midwifery Stud.

[5] Al-Amoudi WM (2017). Protective effects of fennel oil extract against sodium valproate-induced hepatorenal damage in albino rats. Saudi J Biol Sci.

[6] Amirghofran Z, Azadbakht M, Keshavarzi F (2000). Echium amoenum stimulate of lymphocyte proliferation and inhibit of humoral antibody synthesis. Iran J Med Sci.

[7] Amirghofran Z (2010). Medicinal plants as immunosuppressive agents in traditional Iranian medicine. Iran J Immunol.

[8] Appleton SM (2018). Premenstrual syndrome: evidence-based evaluation and treatment. Clin Obstet Gynecol.

[9] American Psychiatric Association (2013). Diagnostic and Statistical Manual of Mental Disorders, 5th ed.

[10] Bakhtiar L, Gruner OC, Shah MH, Crook JR (1999). The Canon of Medicine of Avicenna. The Law of Natural Healing : Pharmacopia.

[11] Azam Khan M (2004). Aksir Azam.

[12] Azizi H, Ghafari S, Ghods R, Shojaii A, Salmanian M, Ghafarzadeh J (2018). A review study on pharmacological activities, chemical constituents, and traditional uses of Echium amoenum. Phcog Rev.

[13] Babazadeh R, Keramat A (2011). Premenstrual syndrome and complementary medicine in Iran: a systematic review. Feyz.

[14] Bawazir A, Bokhary LE (2016). Study the effect of foeniculum vulgare mill seeds essential oil on some neurotransmitters content and histological structure of cerebellar cortex in male albino rats. Adv Environ Biol.

[15] Bekhradi R, Zafari M (2012). Comparing the effects of vitamin E and fennel extract on intensity of primary dysmenorrhea. J Mazandaran Univ Med Sci.

[16] Bieber EJ, Sanfilippo JS, Horowitz IR, Shafi MI (2015). Clinical gynecology.

[17] Bryant M, Cassidy A, Hill C, Powell J, Talbot D, Dye L (2005). Effect of consumption of soy isoflavones on behavioural, somatic and affective symptoms in women with premenstrual syndrome. Bri J Nutr.

[18] Chiaramonte D, Ring M, Locke AB (2017). Integrative women’s health. Med Clin.

[19] Delaram M, Heydarnejad MS (2011). Herbal remedy for premenstrual syndrome with fennel (foeniculum vulgare)–randomized, placebo-controlled study. Adv Clin Exp Med.

[20] Delaram M, Sadeghiyan Z, Jafari F, Khairi S, Bekhradi E, Rafeiyan M (2011). Comparison of effect of echinophora-platyloba, fennel and placebo on pre-menstural syndrome in Shahre Kord university students. SSUJ.

[21] Dutta A, Sharma A (2021). Prevalence of premenstrual syndrome and premenstrual dysphoric disorder in India: a systematic review and meta-analysis. Health Prom Perspect.

[22] Duvan CI, Cumaoglu A, Turhan NO, Karasu C, Kafali H (2011). Oxidant/antioxidant status in premenstrual syndrome. Arch Gynecol Obstet.

[23] Endicott J, Nee J, Harrison W (2006). Daily Record of severity of problems (DRSP): reliability and validity. Arch Womens Mental Health.

[24] Farahmand M, Khalili D, Ramezani Tehrani F, Amin G, Negarandeh R (2020). Effectiveness of echium amoenum on premenstrual syndrome: a randomized, double-blind, controlled trial. BMC Complement Med Ther.

[25] Gama CRB, Lasmar R, Gama GF, Oliveria L, Naliato ECDO, Ribeiro MG, Fonseca ADSD, Geller M (2014). Premenstrual syndrome: clinical assessment of treatment outcomes following Borago officinalis extract therapy  in Patients Presenting with Cyclical Mastalgia. Revista Berasileria de Medicina.

[26] Gnanasambanthan S, Datta S (2019). Premenstrual syndrome. Obstet Gynaecol Reprod Med.

[27] Gold EB, Wells C, Rasor MO (2016). The association of inflammation with premenstrual symptoms. J Womens Health.

[28] Gori L, Gallo E, Mascherini V, Mugelli A, Vannacci A, Firenzuoli F (2012). Can estragole in fennel seed decoctions really be considered a danger for human health? A fennel safety update. Evid Based Complement Alternat Med.

[29] Hooshmand S, Mahdinezhad MR, Taraz Jamshidi S, Soukhtanloo M, Mirzavi F, Iranshahi M, Hasanpour M, Ghorbani A (2021). Morus nigra L. extract prolongs survival of rats with hepatocellular carcinoma. Phytother Res.

[30] Izadi S (2010). Review the effectiveness of women-centered group psychotherapy on premenstrual syndrome and quality of life of female students in Shahid Chamran University. [Thesis in Persian]. Ahvaz.

[31] Karampoor P, Azarnia M, Mirabolghasemi G, Alizadeh F (2014). The effect of hydroalcoholic extract of fennel (foeniculum vulgare) seed on serum levels of sexual hormones in female wistar rats with polycystic ovarian syndrome (PCOS). J Arak Uni Med Sci.

[32] Kazemi N, Esmaielzadeh F, Kasraeian M (2022). Investigating the effect of resistance band training and consuming fennel extract on premenstrual syndrome in Shiraz high school girls. Nursing and Midwifery Journal.

[33] Khayat S, Fanaei H, Kheirkhah M, Moghadam ZB, Kasaeian A, Javadimehr M (2015). Curcumin attenuates severity of premenstrual syndrome symptoms: a randomized, double-blind, placebo-controlled trial. Complement Ther Med.

[34] Mahboubi M (2019). Evening primrose (oenothera biennis) oil in management of female ailments. J Menopausal Med.

[35] Maleki-Saghooni N, Karimi FZ, Moghadam ZB, Najmabadi KM (2018). The effectiveness and safety of Iranian herbal medicines for treatment of premenstrual syndrome: a systematic review. Avicenna J Phytomed.

[36] Miraj S, Kiani S (2016). A review study of therapeutic effects of Iranian borage (echium amoenum fisch). Der Pharmacia Lettre.

[37] Mirghafourvand M, Asghari Jafarabadi M, Ghanbari-Homayi S (2015). Comparison of the diagnostic values of premenstrual syndrome screening tool (PSST) and daily record of severity of problems (DRSP). J Babol Uni Med Sci.

[38] Montazeri A, Goshtasebi A, Vahdaninia M, Gandek B (2005). The short form health survey (SF-36): translation and validation study of the Iranian version. Qual Life Res.

[39] Omidali F (2015). The effect of Pilates exercise and consuming Fennel on pre-menstrual syndrome symptoms in non-athletic girls. cmja.

[40] Ostad S, Soodi M, Shariffzadeh M, Khorshidi N, Marzban H (2001). The effect of fennel essential oil on uterine contraction as a model for dysmenorrhea, pharmacology and toxicology study. J Ethnopharmacol.

[41] Pazoki H, Bolouri G, Farokhi F, Azerbayjani MA (2016). Comparing the effects of aerobic exercise and Foeniculum vulgare on pre-menstrual syndrome. Middle East Fertil Soc J.

[42] Ranjbaran M, Samani RO, Almasi-Hashiani A, Matourypour P, Moini A (2017). Prevalence of premenstrual syndrome in Iran: a systematic review and meta-analysis. Int J Reprod Biomed.

[43] Rapkin AJ, Akopians AL (2012). Pathophysiology of premenstrual syndrome and premenstrual dysphoric disorder. Menopause Int.

[44] Ryu A, Kim T-H (2015). Premenstrual syndrome: a mini review. Maturitas.

[45] Sadock BJ (2007). Kaplan & Sadock's synopsis of psychiatry: behavioral sciences/clinical psychiatry.

[46] Salehi A, Marzban M, Amini F (2019). Effect of foeniculum vulgare on primary dysmenorrhea: a systematic review and meta-analysis. Women Health Bull.

[47] Sayyah M, Boostani H, Pakseresht S, Malaieri A (2009). Efficacy of aqueous extract of Echium amoenum in treatment of obsessive–compulsive disorder. Prog Neuro-Psychopharmacol Biol Psychiatry.

[48] Sayyah M, Siahpoosh A, Khalili H, Malayeri A, Samaee H (2012). A double-blind, placebo-controlled study of the aqueous extract of Echium amoenum for patients with general anxiety disorder. Iran J Pharm Res.

[49] Seidlova-Wuttke D, Wuttke W (2017). The premenstrual syndrome, premenstrual mastodynia, fibrocystic mastopathy and infertility have often common roots: effects of extracts of chasteberry (vitex agnus castus) as a solution. Clinical Phytoscience.

[50] Hugh S, Lubna Pal (2019). Speroff's clinical gynecologic endocrinology and infertility.

[51] Torkzahrani S, Akhavan-Amjadi M, Mojab F, Majd HA (2007). Clinical effects of Foeniculum vulgare extract on primary dysmenorrhea. J Reprod Infertil.

[52] Watanabe S, Sakurada M, Tsuji H, Matsumoto S, Kondo K (2005). Efficacy of γ-linolenic acid for treatment of premenstrual syndrome, as assessed by a prospective daily rating system. J Oleo Sci.

[53] Zendehdel M, Elyasi F (2018). Biopsychosocial etiology of premenstrual syndrome: a narrative review. J Family Med Prim Care.

